# Novel Millet-Based Flavored Yogurt Enriched With Superoxide Dismutase

**DOI:** 10.3389/fnut.2021.791886

**Published:** 2022-01-04

**Authors:** Xiankang Fan, Xiefei Li, Tao Zhang, Yuxing Guo, Zihang Shi, Zhen Wu, Xiaoqun Zeng, Daodong Pan

**Affiliations:** ^1^Key Laboratory of Animal Protein Food Processing Technology of Zhejiang Province, College of Food and Pharmaceutical Sciences, Ningbo University, Ningbo, China; ^2^State Key Laboratory for Managing Biotic and Chemical Threats to the Quality and Safety of Agro-Products, Ningbo University, Ningbo, China; ^3^School of Food Science and Pharamaceutical Engineering, Nanjing Normal University, Nanjing, China

**Keywords:** *lactic acid bacteria*, superoxide dismutase, yogurt, HPLC-MS, HS-SPME-GC-MS

## Abstract

Superoxide dismutase (SOD) is an important antioxidant enzyme with different physiological functions, which can be used as a nutritional fortifier in food. Cereal-based fermented products are becoming popular worldwide. In this study, novel millet-based flavored yogurt enriched with SOD was developed. *Lactiplantibacillus plantarum subsp. plantarum* was screened, which manufactured SOD activity of 2476.21 ± 1.52 U g^−1^. The SOD content of millet yogurt was 19.827 ± 0.323 U mL^−1^, which was 63.01, 50.11, and 146.79% higher than that of Bright Dairy Yogurt 1911, Junlebao and Nanjing Weigang, respectively. Fifty-four volatile flavor substances and 22,571 non-volatile flavor substances were found in yogurt. Compared to traditional fermented yogurt, 37 non-volatile metabolites in yogurt with millet enzymatic fermentation broth were significantly upregulated, including 2-phenyl ethanol, hesperidin, N-acetylornithine and L-methionine, which were upregulated by 3169.6, 228.36, 271.22, and 55.67 times, respectively, thereby enriching the sensory and nutritional value of yogurt. Moreover, the manufacture of unpleasant volatile flavor substances was masked, making the product more compatible with consumers' tastes.

**Graphical Abstract:**
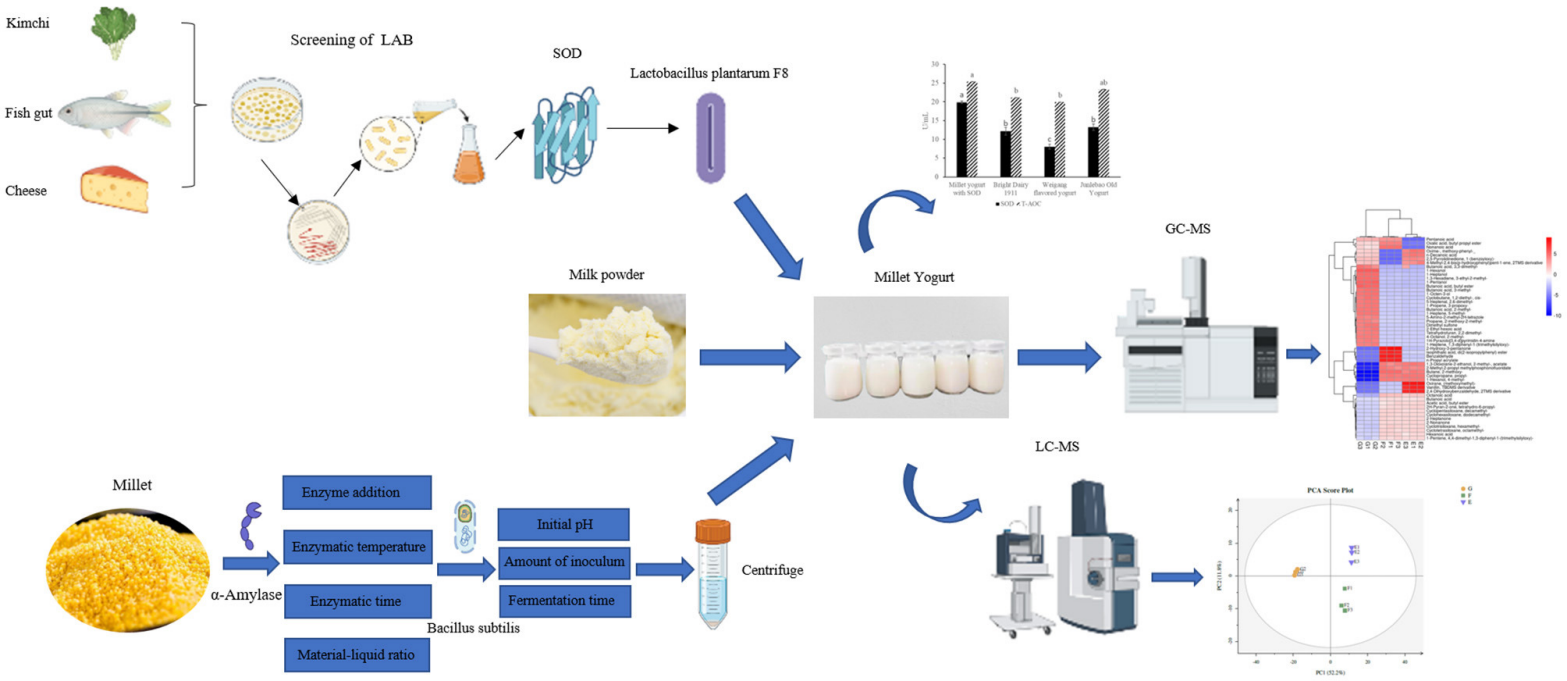
Novel millet-based flavored yogurt enriched with superoxide dismutase. Simplified experimental protocol for the preparation of SOD-enriched millet flavored yogurt.

## Introduction

The probiotic industry has embarked on a new windfall in the post-epidemic era, with market size of $51.2 billion in 2020, and the global probiotic market is expected to grow at a CAGR of 6.9% from 2020 to 2025, while probiotic yogurt accounts for about 78% of the total global probiotic business (1, 2). Yogurt is a traditional dairy product manufactured by *Lactobacillus delbrueckii subsp. bulgaricus* and *Streptococcus thermophilus* by lactic acid fermentation (3). The direction of yogurt development is to develop high quality yogurt enriched with functional factors (4). The fermentation of *Lactobacillus* (LAB) manufactures superoxide dismutase (SOD) and peptides, which make yogurt richer in nutrients, easier to be digested and absorbed by the body (5).

SOD could scavenge superoxide anion free radicals and has various effects, such as anti-aging and anti-inflammatory. Therefore, SOD exhibited various applications in health care products, food and medicine (6). Many scholars have calculated the SOD content in microorganisms, and the results indicate that SOD is widespread in microorganisms. Therefore, the purpose of this study was to screened a strain of LAB with high SOD production to enhance the antioxidant function of yogurt. Some studies have found that using LAB with antioxidant activities can improve the antioxidant function of fermented products. After fermentation using the *Lactiplantibacillus plantarum* BCRC 10357, the total phenols and flavonoids in stone lotus increased, leading to improve the superoxide factor, DPPH free-radical scavenging ability, and the T-AOC of stone lotus (7). Soy milk fermented using *Lacticaseibacillus rhamnosus* CRLP81 has a higher T-AOC than the unfermented one, and it can inhibit DNA oxidative damage (8). Clinical studies have discovered that compared with the control group, the SOD activity in the serum of volunteers who ate kelp fermented with *Lactobacillus sp*. BJ20 is higher (9).

Fruit or plant ingredients are often added to yogurt to add sweetness and nutritional value, a practice that can also counteract the natural sourness of the yogurt. For example, pumpkin has been frequently used as a functional food for its antioxidant, antitumor, immunomodulatory, hypoglycemic and hepatoprotective properties (10). Millet contains some active antioxidant substances, such as minerals, vitamins and phenolic compounds (11). Adding millets to acid whey can be used to neutralize acid whey and enable efficient upcycling (12). Xiao Song reported the effect of polymerized whey protein and xanthan gum as thickeners of millet yogurt products on gel and microstructure qualities (13). However, the effect of millet enzymatic fermentation broth on the flavor and antioxidant capacity of yogurt was rarely reported. The treatment of millet was usually done through enzymatic digestion, but it has been improved by inoculating the millet with *Bacillus subtilis* after enzymatic digestion for further fermentation to enhance its utilization and nutritional value in this study. *Bacillus subtilis* is an essential biological leavening agent, many countries and regions use it to make traditional fermented legumes, such as Chinese tempeh and Japanese natto (14). Fermentation of millet enzymatic digests using *Bacillus subtilis* can improve its antioxidant capacity. According to reports, red bean products fermented using *Bacillus subtilis* IMR-NK1 exhibited significant antioxidant activity (15). The fermentation of black soybeans using *Bacillus subtilis*, which can improve the content of total phenols and isoflavones in the fermentation broth, can enhance its antioxidant activity (16). Therefore, we used *Bacillus subtilis* 109047 to ferment millet enzymatic solution, expecting to improve its antioxidant capacity as well as nutritional value.

However, yogurt will eventually be sold to the public, so the senses of yogurt are very crucial. The addition of high SOD-producing LAB and millet fermentation might affect the flavor of yogurt, so it was necessary to detect the flavor. Most of the flavor compounds in yogurt are manufactured by milk fat lipolysis and the microbial conversion of lactose and citrate (17). More than 100 volatile flavors were found in yogurt, including carbonyl compounds, alcohols, acids, esters, hydrocarbons and aromatic compounds (3). HS-SPME-GC-MS and HPLC-MS can be used to determine volatile and non-volatile flavor substances in yogurt, respectively. Erkaya et al. have detected 34 volatile compounds using HS-SPME-GC-MS in yogurt made from the milk of sheep, cows, goats and buffaloes, of which acetaldehyde, diacetyl and acetylene were the major volatile compounds (18). A total of 196 non-volatile metabolites, including nucleosides, amino acids, carbohydrates and lipids, have been evaluated using UPLC-Q-TOF-MS (19).

This paper aimed to develop a millet-flavored yogurt enriched with SOD. Firstly, a LAB strain with high SOD production was screened, and its probiotic properties were investigated. Then, a millet enzyme fermentation broth was prepared and compounded with milk to develop SOD-rich millet yogurt. Finally, the effects of co-fermentation of screened strains with conventional fermenters on metabolites in yogurt and the effects of millet enzymatic fermentation broth on volatile and non-volatile flavor substances in yogurt were analyzed by metabolomics.

## Materials and Methods

### Materials

Kimchi, fish sausage and cheese, the ingredients used to screen for LAB, were purchased from the Nanjing Farmers' Market. Analysis reagents, including methanol, acetonitrile, methyl tert-butyl ether, formic acid and ammonium formate, were all purchased from Thermo. T-AOC kits and peptide assay kits were purchased from Solarbio.

### Methods

#### Screening of LAB With High SOD Production

LAB was isolated from kimchi, fish sausage and cheese by spreading sample diluent on MRS medium that contained bromocresol purple indicator. The pyrogallol autotrophic method was used to determine the SOD enzyme activity of LAB. The strains were identified by 16S rDNA sequencing (20).

#### Probiotic Potential Evaluation of LAB

The LAB was respectively inoculated into MRS with pH 2.5–8.5 and cultured for 24 h to observe the growth of the bacteria. The activated LAB was inoculated to MRS media with different NaCl content, with NaCl content of 1–8% (w/v) and cultured for 16 h at 37°C to determine their anti-permeable pressure. The LAB was cultured in MRS at pH 7 for 12 h to observe and evaluate the acid production capacity, and cultured in intestinal juice for 8 h and then in gastric juice for 3 h to calculate the survival rate (21). The LAB was successively passaged for 16 generations to determine the genetic stability, and the SOD production ability of the LAB was measured every two generations.

#### Preparation and Optimization of Millet Enzymatic Hydrolysis Fermentation Broth

The millet powder with a particle size of 80 mesh was prepared, mixed with water and heated at 80°C for 60 min. The gelatinized millet powder was liquefied by alpha-amylase and then boiled. The supernatant was obtained and used to determine the T-AOC and dextrose equivalent (DE) after the gelatinized millet powder had been centrifuged at 8000 ×g for 10 min at 20°C. The solid-liquid ratio (A), with enzyme amount (B), enzymatic digestion time (C) and enzymatic digestion temperature (D) were selected as four factors might affect the quality of millet enzymatic digestion solution for single-factor experiments.

#### Changing Different Enzymolysis Conditions and the Optimizing Enzymolysis Scheme

The millet enzymatic hydrolysate was fermented with *Bacillus subtilis*109047 at 37°C for a specific period, and then the T-AOC was determined.


$$\begin{array}{l} \text{T}-\text{AOC }\left(\text{U}/\text{mL}\right)\;=\left(\text{ x}\times \text{V}1\right)÷\text{V}2\;=\;34\times \text{x} \end{array}$$

Where V1 represents the total volume of the reaction (0.204 mL); V2 represents the volume of the sample in the reaction (0.006 mL), and x is calculated based on the standard curve.


$$\begin{array}{l} DE\;\left(\%\right)\;=\;\left(C\times VT\right)÷\left(m\times VS\right)\times 100 \end{array}$$

Where C represents the amount of sugar (mg) based on the standard curve; VT represents the volume of the extract; m represents the mass of the sample, and VS represents the volume (mL) of the sample used in the determination.

#### Development of Millet Yogurt Rich in SOD

The enzymatic fermentation broth of millet was centrifuged at 8,000 ×g, 4°C for 10 min. The supernatant was mixed with recovered milk and sterilized in a water bath at 95°C for 10 min, then cooled in a water bath at 42°C. Yogurt fermenter (*Lactobacillus delbrueckii subsp. bulgaricus and Streptococcus thermophilus*) with high SOD-producing LAB were inoculated into the above 42°C cooled recovered milk. The recovered milk was homogenized in a sterile environment and then fermented in an incubator at 42°C for 6 h (22). The yogurt with the most SOD content and best sensory were prepared by adding different amounts of starter, using different material-to-liquid ratios, adding different amounts of sucrose, adding phytochemicals and using different fermentation times.

#### Sensory Evaluation

The sensory evaluation was approved by the Ningbo University Institutional Review Board (NU-IRB). According to Mengdi Yin, sensory evaluation was performed using the quantitative descriptive analysis (QDA) method (23). The sensory evaluation was conducted by a professionally trained panel of 15 males and 15 females. The examined yogurt samples took into account four individual quality characteristics, including organization status, taste, odor and color. As shown in Table 1, the descriptors describing the characteristics of the examined samples and their definitions were decided by the panel after the training.

**Table 1:** Sensory rating scale.

**Items**	**Evaluation criteria**	**Score**
Organizational status	Yogurt has uniform texture, moderate viscosity and no whey precipitation.	20–25
	The yogurt has a relatively uniform texture, moderate viscosity and a small amount of whey precipitation.	15–20
	Yogurt has an uneven texture, low viscosity, and more whey precipitation.	0–15
Taste	The yogurt is moderately sweet and sour, delicate and smooth, and easily accepted.	20–25
	The yogurt is out of proportion to the sweet and sour, and the taste is a little rough but acceptable.	15–20
	Yogurt tastes too sour or too sweet and is not easy to accept.	0–15
Odor	It has the inherent whey flavor and pleasant odor of yogurt.	20–25
	Yogurt has an inherent milky flavor that is relatively light and slightly sour.	15–20
	Yogurt has no creamy flavor, heavy sourness and incongruous odor.	0–15
Color	The yogurt is creamy white and shiny, uniform in color and free of impurities.	20–25
	The yogurt is creamy and slightly shiny, dark in color and free of impurities.	15–20
	Yogurt is milky white and lusterless, dull in color, with impurities.	0–15

#### Determination of SOD Content in Yogurt

The pH of the yogurt was adjusted to 4.6 with 2 mol L^−1^ H_3_PO_4_, and then the yogurt was bathed in water at 38°C for 5–10 min. After the treated yogurt was centrifuged at 7,000 ×g for 10 min, the supernatant was removed and adjusted to pH 6.0 with 0.5 mol L^−1^ NaOH for subsequent SOD content test.

#### Evaluation of Various Indicators of Yogurt

The pH value of the final yogurt and the content of LAB was measured using a pH meter and the dilution coating method. The differences in T-AOC and peptide content among the final yogurt and Bright Dairy 1911, Junlebao and Nanjing Weigang flavor yogurt were compared.

#### Analysis of Non-volatile Flavor Substances in Yogurt by HPLC-MS

The yogurt was fermented in a 42°C incubator. The types of yogurts were as follows: traditional starter (Y), traditional starter and *Lactiplantibacillus plantarum subsp. plantarum* F8 (Y-F8), and millet yogurt (Y-F8-M). Non-targeted metabolomics was used to analyze the effect of the addition of *Lactiplantibacillus plantarum subsp. plantarum* F8 and millet enzymatic fermentation broth on non-volatile flavor substances in yogurt (23–25). Metabolites were initially extracted from yogurt samples. Then, all samples were thawed at 4°C, 100 mg of which was transferred into 2 mL centrifuge tubes, added with 200 μL of methanol and 200 μL of MTBE into each centrifuge tube, and shaken for 60 s. Afterward, the samples were centrifuged at 4°C for 10 min at 12,000 rpm, and the supernatant was filtered through a 0.22 μm membrane to obtain the prepared samples for HPLC-MS (Thermo U300; QE). The chromatographic conditions were as follows: a 1.8 μm (2.1 × 150 mm) chromatographic column was used; injection port temperature of 8°C; flow rate of 0.25 mL min^−1^; column temperature of 40°C; and a 2 μL injection was used for gradient elution. The mobile phase was positive ion 0.1% formic acid water 0.1% formic acid acetonitrile and negative ion 5 mM ammonium formate water acetonitrile. Finally, a mass spectrometer was used an electrospray ionization source in positive and negative ionization mode with positive and negative ion spray voltages of 3.50 and 2.50 kV, respectively, a sheath gas of 30arb, and an auxiliary gas of 10 arb. The capillary was set at 325°C, and a full scan was performed with a resolution of 70,000, a scan range of 81–1,000, and a secondary cleavage with HCD, with a collision voltage of 30 eV (19, 26, 27).

#### Analysis of Volatile Flavor Compounds in Yogurt by HS-SPME-GC-MS

The HS-SPME device was used to extract volatile flavor compounds in yogurt for 60 min at 50°C and 350 rpm. The GC-MS instrument make and model was Agilent 8890 GC System+5977B/MSD. The chromatographic conditions were set at an initial temperature of 35°C for 3 min, 4°C min^−1^ to 140°C for 1 min, and 10°C min^−1^ to 250°C for 3 min. The injection temperature was set at 250°C; the carrier gas was He, and the flow rate was 1.0 mL min^−1^. The mass spectrometry conditions were as follows: ionization mode EI of 70 eV, ion source temperature of 230°C, mass scanning range of 33–650 AMV, and emission voltage of 100 μA (22, 28). Agilent MssHunter Qualitative Analysis B.07.00 software and NIST14.LMS database provided by Agilent were used for data comparison, while we referred to metabolomics analysis methods from other scholars (29–32).

#### Statistical Analysis

Differences between samples were analyzed using one-way analysis of variance (ANOVA) and Duncan's multiple range tests using SPSS. A *P*-value of <0.05 was considered to be statistically significant. All experiments were conducted three times in parallel. Bioinformatics analyses such as PCA plots and heat maps were performed using the OmicStudio tool at https://www.omicstudio.cn/tool.

## Results and Discussion

### Screening of LAB With High SOD Production

**Figure 1:**
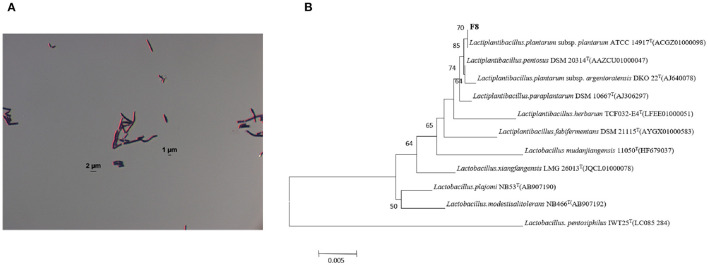
**(A)** Results of Gram staining of *Lactiplantibacillus plantarum subsp. plantarum F8*. **(B)** Phylogenetic tree of 16S rDNA sequence of the target strain *Lactiplantibacillus plantarum subsp*. *plantarum F8* and related species.

Twenty-one strains of LAB were screened and their SOD enzyme activities were determined. The results were indicated in Table 2, a target strain with high SOD production was screened, and the SOD content of this strain reached 2476.21 ± 1.52 U g^−1^. This strain had the highest SOD content among the 21 strains of LAB. Based on the 16S rDNA sequence, the screened F8 strain was identified as: *Lactiplantibacillus plantarum subsp. plantarum* (Figure 1).

**Table 2:** SOD content of *lactic acid bacteria* screened.

**Bacteria number**	**SOD (U/g)**	**Bacteria number**	**SOD (U/g)**
F1	597.48 ± 2.74	F12	2143.97 ± 0.68
F2	363.07 ± 3.68	F13	854.29 ± 6.71
F3	731.47 ± 2.18	F14	1238.99 ± 9.56
F4	682.85 ± 2.25	F15	871.56 ± 2.32
F5	1379.85 ± 1.93	F16	1358.69 ± 7.21
F6	659.46 ± 3.37	F17	587.11 ± 5.79
F7	489.44 ± 3.09	F18	607.05 ± 7.91
F8	2476.21 ± 1.52	F19	563.78 ± 4.23
F9	487.54 ± 4.33	F20	454.26 ± 3.56
F10	963.44 ± 4.37	F21	284.55 ± 5.44
F11	1594.24 ± 5.05		

*SOD, Superoxide dismutase*.

**Figure 2:**
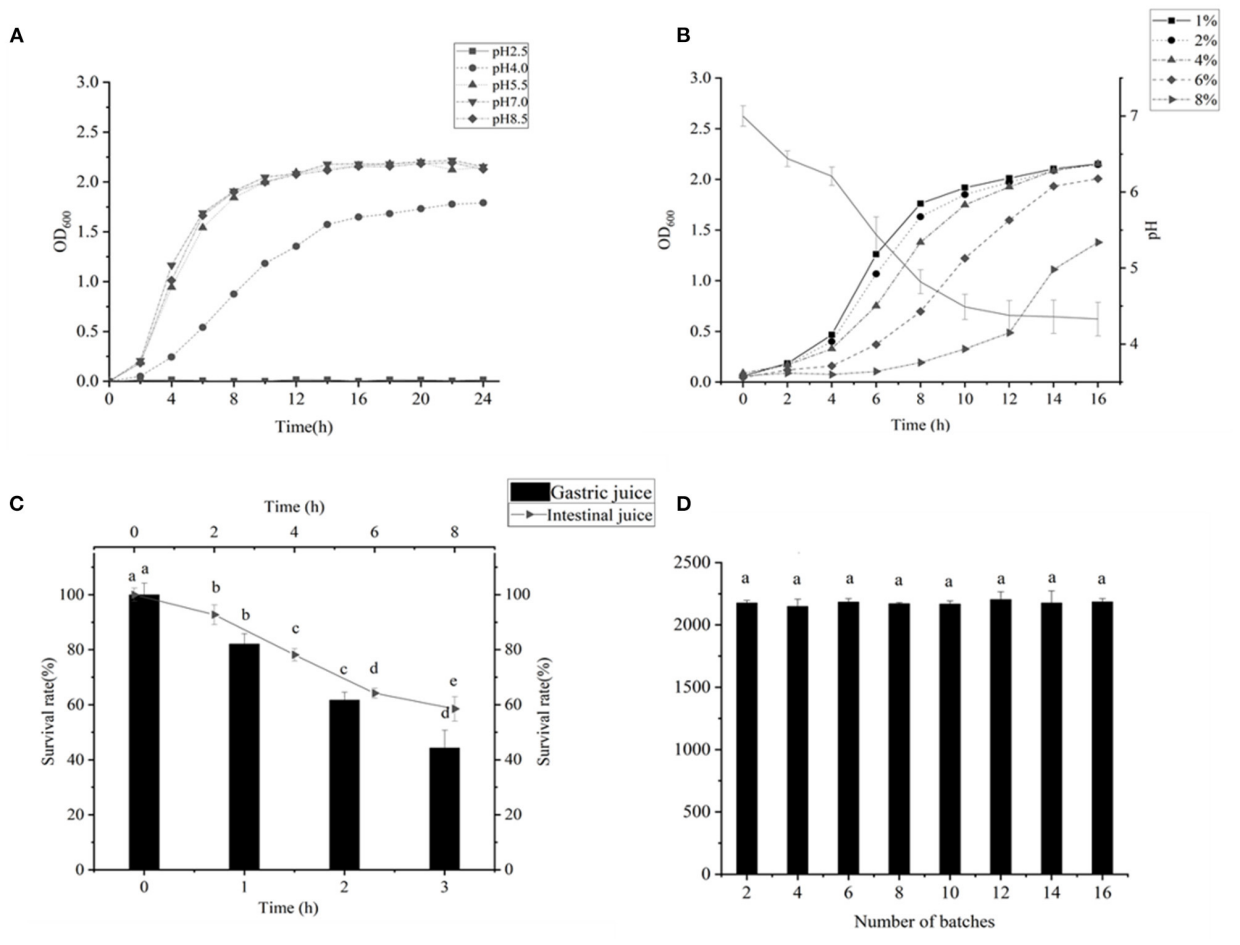
**(A)** Result of the acid resistance test of *Lactiplantibacillus plantarum subsp*. *plantarum F8*. **(B)** Determination of acid production capacity; the pH of the medium reduced from 7 to 4.38 within 12 h; 1–8% NaCl was added to the MRS medium, and the growth of the bacteria was measured in 2–16 h. **(C)** Survival rate of LAB in artificial intestinal juice within 8 h. Survival rate of LAB in artificial intestinal juice for 3 h. **(D)** LAB were successively passaged for 16 generations, and the SOD content was measured every two generations. The genetic performance was stable. Significant differences were indicated by the letters a, b and c. Any difference with the same letter was considered not significant (*p* > 0.05) and any difference with a different letter was considered significant (*p* < 0.05).

As indicated in Figure 2A, the LAB grew well at a pH of 4, and the LAB needed good acid resistance to maintain their activity in a gastric acid environment (33). Figure 2B shows that the bacteria could decrease the pH of the fermentation broth to 4.38 within 12 h, which indicated the high acid production capacity of this LAB. This was close to the results of A H X G screening LAB for fermentation of high-quality yogurt (34). The strain could withstand the osmotic pressure of 8.0% sodium chloride, indicating that the strain might also have application prospects in fermented kimchi and other low-salt pickled foods. The prerequisite that LAB could play a beneficial role in the intestine is to be able to tolerate the gastrointestinal environment and survive (31). As shown in Figure 2C, the bacterium exhibited very good tolerance in gastrointestinal fluids, reaching 58.54% survival in intestinal fluids for 8 h and 61.7% survival in gastric fluids for 2 h. This is similar to the result of Wu et al. (35). In addition, the bacterium exhibited excellent genetic stability of SOD (Figure 2D), which is similar to Chooruk A's results (36).

### Preparation and Optimization of Millet Enzymatic Hydrolysis Fermentation Broth

**Figure 3:**
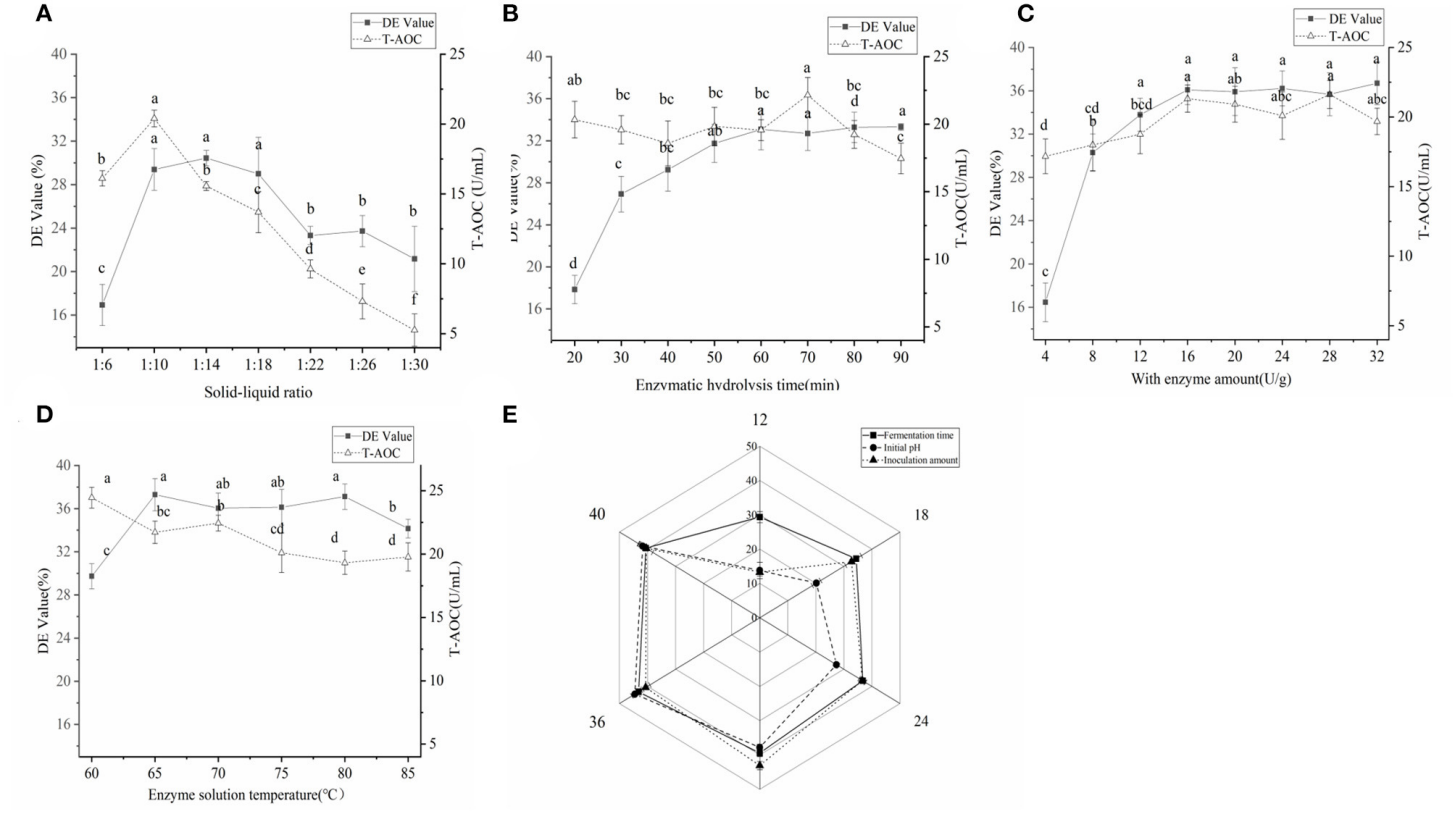
**(A)** The effect of the material-to-water ratio on the quality of the enzymatic hydrolysate; the material-to-water ratio was set to 1:6, 1:10, 1:14, 1:18, 1:22, 1:26, and 1:30. **(B)** The effect of enzymolysis time on the quality of enzymolysis solution; the enzymolysis time was set to 20–90 min. **(C)** Amount of enzyme added. **(D)** The influence of enzymolysis temperature. **(E)** The effect of fermentation time of *Bacillus subtilis* inoculum and initial pH on the enzymatic hydrolysate of the fermentation broth. Significant differences were indicated by the letters a, b and c. Any difference with the same letter was considered not significant (*p* > 0.05) and any difference with a different letter was considered significant (*p* < 0.05).

In the single-factor experiment of millet enzymatic digestion (Figures 3A–D), the best enzymatic digestion conditions were as follows: solid-liquid ratio(A) of 1:10 (w/v), amylase addition (B) of 16 U g ^−1^, enzymatic digestion time (C) of 70 min and enzymatic digestion temperature (D) of 65°C. Based on the orthogonal test results (Table 3), the order of factors affecting the DE value of millet enzymatic hydrolysate was D>B>C>A. When the T-AOC was used as the standard, the order of factors affecting the T-AOC of millet enzymatic hydrolysate was A>C>B>D. The best combination was obtained from A_1_B_2_C_1_D_3_. Considering these two factors, the best combination was determined as A_1_B_1_C_3_D_1_, which is, the ratio of the material to liquid was 1:10 (w/v). The amount of enzyme added was 16 U g^−1^, the enzymatic hydrolysis time was 80 min and the enzymatic hydrolysis temperature was 65°C. Three repetitive experiments were conducted under this condition. The average DE value was 40.03 ± 0.85% and the T-AOC was 26.24 ± 0.53 U mL^−1^. The DE value increased by 7.35% and the T-AOC increased by 19.71%. This result was consistent with previous reports that the increased antioxidant capacity of millet enzymatic hydrolysis might be mainly related to the contain of polyphenols and vitamins as well as SOD contained in millet (11).

**Table 3:** Analysis table of orthogonal test results.

	**Take DE value as standard (%)**				**Take T-AOC as standard (U/mL)**			
K_1_	110.81	112.41	108.27	114.39	77.03	71.33	72.59	70.48
K_2_	109.86	112.20	108.87	100.92	73.61	72.24	70.09	71.26
K_2_	108.48	104.61	112.08	113.91	62.73	69.80	70.69	71.63
k_1_	36.94	37.47	36.09	38.13	25.68	23.78	24.20	23.49
k_2_	36.62	37.40	36.29	33.64	24.54	24.08	23.36	23.75
k_3_	36.16	34.87	37.36	37.97	20.91	23.27	23.56	23.87
*R*	0.78	2.60	1.27	4.49	4.77	0.81	0.84	0.38
Excellent level	A_1_	B_1_	C_3_	D_1_	A_1_	B_2_	C_1_	D_3_

*DE, Dextrose Equivalent; T-AOC, total antioxidant capacity*.

As indicated in Figure 3E, with the extension of fermentation time, the T-AOC of millet enzymatic hydrolysis fermentation broth initially increased and then reduced, reaching the maximum at 36 h. The T-AOC of the fermentation broth continued to rise with the increase in the inoculation amount. When the inoculum amount was 3%, the T-AOC of the fermentation broth was the largest. With the increase of pH, the T-AOC increased and then decreased, reaching a maximum value of 44.53 ± 1.08 U mL^−1^ at pH 7. As the fermentation time was prolonged, the content of *Bacillus subtilis* secreted protease, and other enzymes increased gradually. Large proteins were hydrolyzed into peptides and anti-oxidant substances such as flavonoids and polyphenols, which were found in the millet enzymatic hydrolysate (37). Therefore, it might be that the increase in these antioxidants was what made the T-AOC increased. Afterward, some active peptides were continuously hydrolyzed into free amino acids (38). As time increased, flavonoids and polyphenols continued to decrease, and the T-AOC declined. The amount of inoculation greatly influenced the primary and secondary metabolism of microorganisms (34). When the inoculate volume was at a low level, the enzyme and peptide content in the fermentation broth increased as inoculate volume increases, causing an increase in T-AOC. If the bacteria were cultured in high density, the microorganism's growth and reproduction will be inhibited. Therefore, the enzyme manufactured was relatively small, and the T-AOC was reduced. Therefore, the optimal fermentation conditions were as follows: fermentation time of 36 h, *Bacillus subtilis* addition amount of 3%, and initial pH of 7. Compared with initial fermentation, the T-AOC of the fermentation broth has increased by 69.7%. *Bacillus subtilis* might improve the nutritional value and antioxidant capacity of millet enzymes by producing nattokinase, SOD, and catalase (37).

### Development of Millet Yogurt Rich in SOD

**Figure 4:**
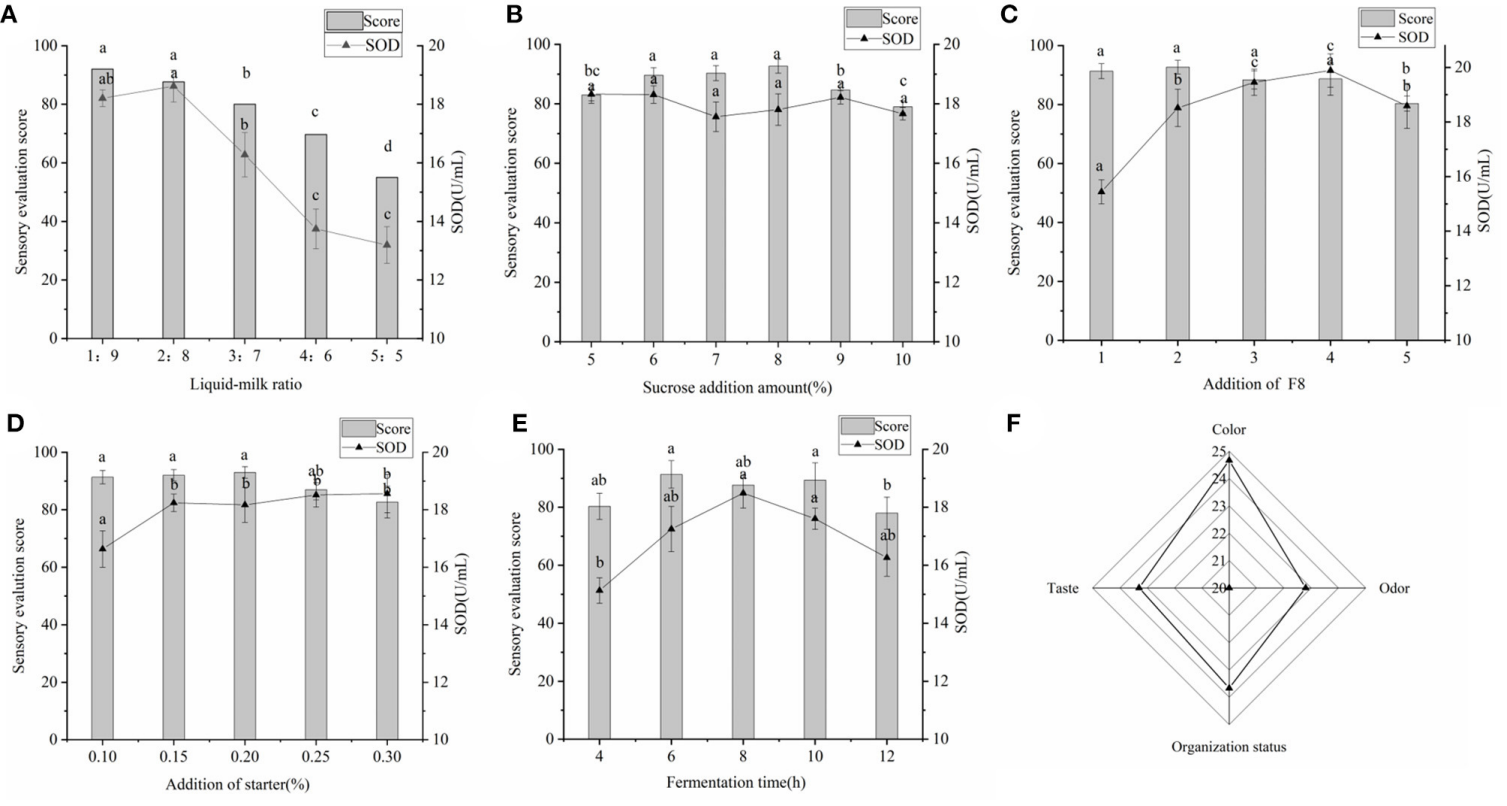
**(A)** Effects of raw material ratio on SOD content and sensory. The ratio of millet enzymatic fermentation broth and fresh milk was set to 1:9, 2:8, 3:7, 4:6, and 5:5. **(B)** Effects of added sucrose on SOD content and sensory. The amount of sucrose added was set to 5–10%. **(C)** Effects of the amount of *Lactiplantibacillus plantarum subsp*. *plantarum F8* on SOD content and sensory sense. **(D)** The effect of direct-input starter on the yogurt quality. **(E)** The influence of fermentation time on yogurt. **(F)** Sensory evaluation chart of final yogurt. Significant differences were indicated by the letters a, b and c. Any difference with the same letter was considered not significant (*p* > 0.05) and any difference with a different letter was considered significant (*p* < 0.05).

During the fermentation of yogurt, about 20% of sugar and protein was broken down into small molecules and fatty acids, which improved the utilization of nutrients. In addition to retaining all the nutrients of yogurt, lactic acid, amino acids and fatty acids produced by LAB were easily absorbed by the body (39). The SOD content in yogurt reached the maximum at 2:8 (v/v) (Figure 4A), and at this time the yogurt had moderate viscosity, no whey precipitation, special yogurt taste, creamy white color and glossy. The SOD content in yogurt reached the maximum at 2:8 (v/v), and at this time the yogurt had moderate viscosity, no whey precipitation, special yogurt taste, creamy white color and glossy. The addition of sucrose had almost no effect on the SOD content in yogurt, but had a great influence on the sensory evaluation score of yogurt taste (Figure 4B), which is consistent with previous reports (6).

The SOD content increased significantly with increasing inoculum, reaching a maximum at 4% (v/v) (Figure 4C), indicating that the addition of LAB with high SOD production might affect the SOD content of yogurt. This was similar to the results of Wang et al. who fermented stone lotus with *Lactiplantibacillus plantarum* BCRC 10357 (7). As shown in Figure 4D, the SOD content gradually increased with the increase of yogurt fermenters, but there was no significant difference. This might be due to the fact that for yogurt fermenters were composed of *Lactobacillus delbrueckii subsp. bulgaricus and Streptococcus thermophilus*, which could also produce small amounts of SOD during fermentation (22). The SOD content in yogurt reached a maximum at 8 h (Figure 4E), after which it gradually decreased. This could be explained by the gradual decrease in pH with increasing fermentation time, which might affect the SOD production and SOD enzyme activity of LAB (6).

To obtain the best recipe for making millet yogurt, an orthogonal test was conducted. Based on the orthogonal results (Table 4), the order of influence on the SOD content in millet yogurt was A>B>D>C. The optimal conditions were as follows: a liquid-to-milk ratio of 2:8 (v/v), amount of added *Lactiplantibacillus plantarum subsp. plantarum* F8 of 3% (v/v), amount of added direct starter of 0.25% (w/v), and fermentation time of 6 h. Re-experiments verified that the SOD content in yogurt was 19.827 ± 0.323 U mL^−1^, which was increased by 7.25% compared with the previous orthogonal optimization. Feng et al. determined the SOD content in human and certain livestock milk and found that sow milk, dog milk, human milk and cow milk were 69.6, 34.9, 7.1, and 3.2 U mL^−1^, respectively (40). By contrast, the SOD content of the millet yogurt developed in this experiment was similar to that of dog milk, and it was much higher than that of human milk and cow milk. SOD is the only enzyme known to directly scavenge free radicals, breaking down superoxide dismutase in organisms into oxygen and hydrogen peroxide with high specificity and efficiency (6). Overexpression of SOD has also been shown to mitigate radiation-induced damage (41). The SOD-rich millet yogurt we have developed was expected to appear in the recipes of people with pre-cancer or to prevent the development of diseases such as cancer.

**Table 4:** L_9_(4^3^) Orthogonal results of millet yogurt rich in SOD.

**Level**	**Experimental factors**				**Results**
	**A**	**B**	**C**	**D**	**SOD (U/mL)**
1	1	1	1	1	16.720 ± 0.200
2	1	2	2	2	17.515 ± 0.745
3	1	3	3	3	18.621 ± 0.227
4	2	1	2	3	17.523 ± 0.175
5	2	2	3	1	19.827 ± 0.323
6	2	3	1	2	17.634 ± 0.540
7	3	1	3	2	15.317 ± 1.326
8	3	2	1	3	16.486 ± 0.361
9	3	3	2	1	17.012 ± 0.027
K_1_	53.856	49.560	50.840	53.559	/
K_2_	54.984	53.828	52.050	50.466	/
K_3_	48.815	53.267	53.765	52.630	/
k_1_	17.610	16.520	16.946	17.850	/
k_2_	18.328	17.942	17.350	16.822	/
k_3_	16.271	17.755	17.921	17.540	/
*R*	2.057	1.422	0.975	1.031	/
Excellent level	A_2_	B_2_	C_3_	D_1_	/

*SOD, Superoxide dismutase*.

Figure 4F was drawn from the results of three independent yogurt sensory evaluations. The results indicated that the yogurt was of high quality. The final yogurt was creamy white, glossy, with a pleasant milk flavor, uniform texture and moderate sweetness. The color, odor, texture and taste were 24.7, 22.8, 23.7, and 23.3 points, respectively. The results were similar to those of Yin M et al. for the sensory evaluation of yogurt (23). The pH of the final yogurt ranged from 4.1 to 4.3, the amount of LAB in fermented millet yogurt was 1.43 × 10^8^ CFU mL^−1^ > 1 × 10^6^ CFU mL^−1^, and no contamination of miscellaneous bacteria was detected in the yogurt. These were in accordance with the regulations for fermented milk (42). The SOD-rich millet yogurt developed by this project was compared with three types of yogurts available on the market. The results were shown in Table 5. The SOD content of the final yogurt was 63.01, 50.11, and 146.79% higher than the ordinary yogurt available on the market, including Bright Dairy 1911, Junlebao Old Yogurt and Weigang-flavored yogurt. This might be due to the high SOD-production LAB we used, in addition, the SOD contained in the millet enzymatic hydrolysis fermentation broth also promoted the total SOD in yogurt (6). The T-AOC of the final yogurt was 19.86, 8.67, and 27.24% higher than the aforementioned ordinary yogurt, respectively. This might be because millet yogurt contained antioxidant substances such as polyphenols, flavonoids and vitamin E from millet, and antioxidant enzymes such as SOD and catalase from *Bacillus subtilis* (37). The peptide content was slightly higher than Weigang-flavored yogurt and lower than Bright Dairy 1911 and Junlebao Old Yogurt. These results indicated that yogurt co-fermented with millet enzymatic digest has high-quality properties and significant antioxidant potential, and thus has great potential as a new functional food in the food industry.

**Table 5:** Comparison of the contents of various substances in ordinary yogurt.

**Category**	**SOD (U/mL)**	**T-AOC (U/mL)**	**Peptide (mg/mL)**
Millet yogurt with SOD	19.827 ± 0.323^a^	25.331 ± 1.691^a^	1.282 ± 0.049^c^
Bright Dairy 1911	12.163 ± 1.001^b^	21.134 ± 1.591^b^	1.649 ± 0.020^b^
Weigang flavored yogurt	8.034 ± 0.614^c^	19.908 ± 2.593^b^	1.057 ± 0.068^d^
Junlebao Old Yogurt	13.208 ± 0.892^b^	23.311 ± 1.207^ab^	1.881 ± 0.047^a^

*SOD, Superoxide dismutase; T-AOC, total antioxidant capacity. Any difference with the same letter was considered not significant (p > 0.05) and any difference with a different letter was considered significant (p < 0.05)*.

### Analysis of Non-volatile Flavor Substances in Yogurt by HPLC-MS

**Figure 5:**
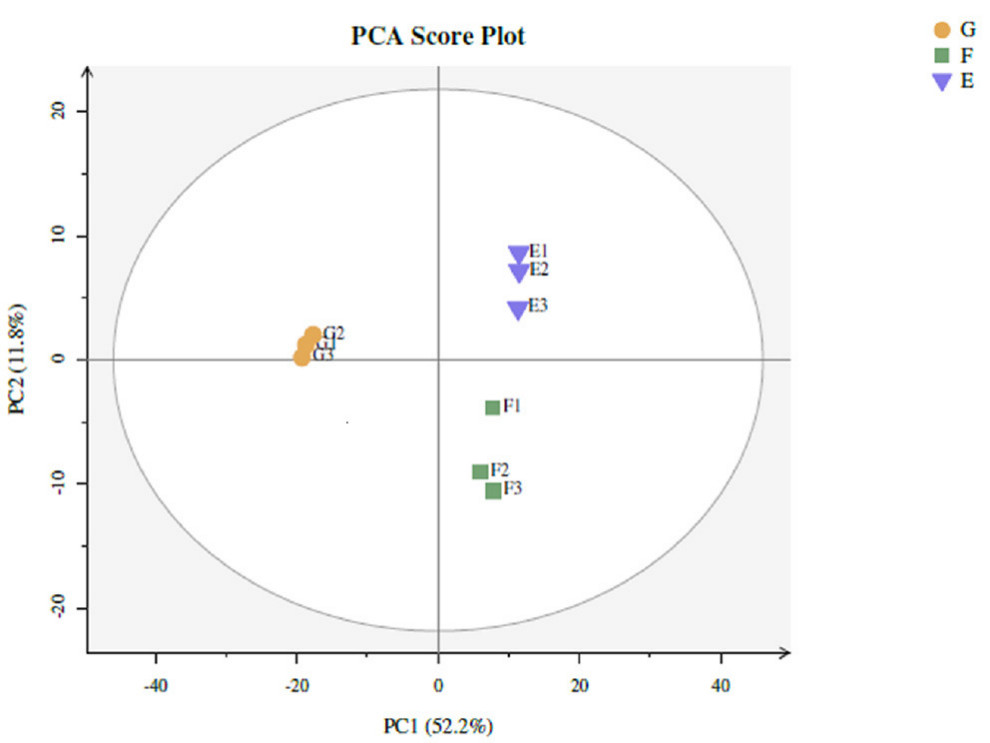
Types of yogurt were as follows: traditional starter (Y), traditional starter and *Lactiplantibacillus plantarum subsp. plantarum F8* (Y-F8), and millet yogurt (Y-F8-M). PCA non-volatile metabolite score for comparison between Y (E), Y-F8 (F), and Y-F8-M (G) (Blue falling triangle: Y, green square: Y-F8, yellow circle: Y-F8-M).

Twenty-two thousand five hundred and seventy-one non-volatile flavor substances from traditional starter (Y), traditional starter and *Lactiplantibacillus plantarum subsp. plantarum* F8 (Y-F8), millet yogurt (Y-F8-M) were screened by LC-MS, and the relevant differences among metabolites were determined. The screening conditions were *p*-value ≤ 0.05, VIP ≥ 1, and Fold_Change ≥ 1.5 or ≤0.667. Two hundred differential metabolites were selected (43). Multivariate statistical analysis was conducted to evaluate non-volatile metabolites and identify any potential variability associated with specific conditions. The PCA score was calculated to determine whether the sample repeatability in the group was sufficiently large (44). As shown in Figure 5, the repeatability of the three yogurt samples was better. The distance of the three yogurt samples was relatively close, the difference differed. The three yogurt samples were separated from the PC1 (52.2%), and along the PC2 direction (11.8%).

**Figure 6:**
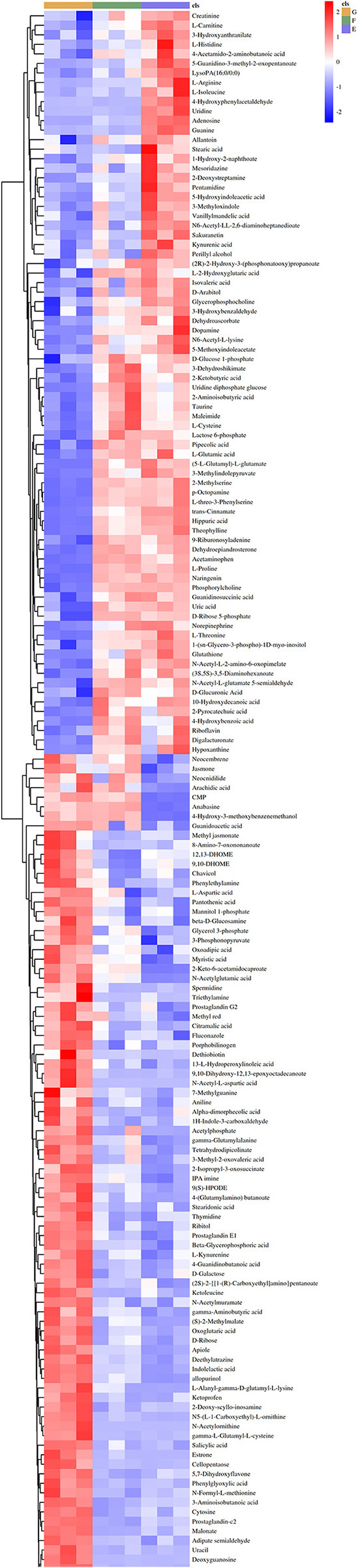
Heat-map and hierarchical clustering of non-volatile metabolite profiles in yogurt. The dendrogram represents sample clusters based on Pearson's correlation coefficient with average linkage. The normalized non-volatile metabolite abundance was visualized by color: red indicates the highest values, and blue indicates the lowest values (E: Y, F: Y-F8, G: Y-F8-M).

**Figure 7:**
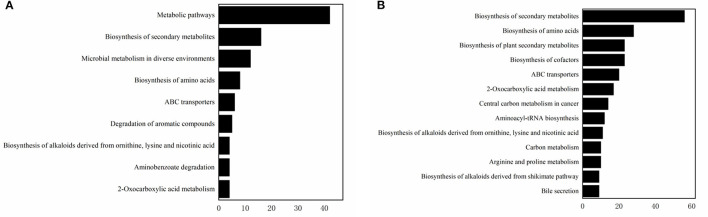
The enriched pathways. **(A)** The enriched pathways involved by significantly increased metabolites when compared group F to group E. **(B)** The enriched pathways involved by significantly increased metabolites when compared group G to group E.

A Heat-map was drawn to distinguish the three groups of sample metabolites (Figure 6). Each small square indicated the metabolic substance content, and the colors indicated the metabolite content. A total of 22,571 non-volatile flavor substances were found in yogurt. Compared with Group E, the change of non-volatile flavor substances in Group F might be mainly related to the 9 metabolic pathways with high significance and high pathway impact in *Lactiplantibacillus plantarum subsp. plantarum* F8. Including 2-Oxocarboxylic acid metabolism, Aminobenzoate degradation, Biosynthesis of alkaloids derived from ornithine, lysine and nicotinic acid, Degradation of aromatic compounds, ABC transporters, Biosynthesis of amino acids, Microbial metabolism in diverse environments and Biosynthesis of secondary metabolites, as shown in Figure 7A. Compared with Group E, 12 metabolites were found to be significant upregulated in group F. Among them, the content of 4-Hydroxy-3-methoxybenzenemethanol was increased by 6.48 times. It had milk, cream and coconut aroma, which was a common baked food natural spice (3). The L-Threonine content was increased 5.45 times, which was considered an essential amino acid with a slightly sweet taste, and it was produced by the action of threonine synthase with high serine phosphate as a substrate (17). The content of allopurinol increased by 4.57 times, which is an isomer of xanthine. It could reduce the production of oxygen free radicals in ischemic re-injection damage to achieve antioxidant effects, so it was often used in primary and secondary hyperuricemia (45). This might also be partly responsible for the high antioxidant capacity of millet yogurt. Arachidonic acid was increased by 2.01 times, and it was a polyunsaturated fatty acid that might play a role in reducing the prevention of diabetes and tumors. In *Lactiplantibacillus plantarum subsp. plantarum*, it was produced by phosphatidylcholine and H_2_O through the arachidonic acid metabolic pathway catalyzed by phospholipase A (46). In addition, nine metabolites were downregulated, with uridine, adenosine and guanine decreasing by 28.57, 4.14, and 4.34 times, respectively. Adenosine is an important intermediate used in the synthesis of adenine, and an increase in purine levels in the body leads to an increase in uric acid, which ultimately leads to hyperuricemia in humans. This was consistent with the reported ability of LAB to absorb and metabolize exogenous nucleosides and purine bases (47). Furthermore, this *Lactiplantibacillus plantarum subsp. plantarum* has a strong nucleoside catabolic capacity and might be applied in the diet of patients with hyperuricemia. The metabolomic analysis indicated that *Lactiplantibacillus plantarum subsp. plantarum* might have a strong probiotic potential and application value.

Compared with Group E, the production of differential non-volatile flavor substances in Group G was related not only to millet enzymatic hydrolysis fermentation broth, but also related to the 13 metabolic pathways with high significance and high pathway impact in *Lactiplantibacillus plantarum subsp. Plantarum* F8. Including Bile secretion, Biosynthesis of alkaloids derived from shikimate pathway, Arginine and proline metabolism, Carbon metabolism, Biosynthesis of alkaloids derived from ornithine, lysine and nicotinic acid, Aminoacyl-tRNA biosynthesis, Central carbon metabolism in cancer, 2-Oxocarboxylic acid metabolism, ABC transporters, Biosynthesis of cofactors, Biosynthesis of plant secondary metabolites, Biosynthesis of amino acids and Biosynthesis of secondary metabolites, as shown in Figure 7B. Compared with Group E, the variety of non-volatile flavor substances in Group G increased by two times, mainly due to the addition of millet enzyme fermentation liquid, which greatly ameliorated the flavor of yogurt. Compared with Group E, the non-volatile flavor substances produced by Group G were increased by 37 and reduced by 44. Among them, 2-Phenylethanol was increased 3169.6 times, a colorless liquid organic matter with a rose aroma (44). The content of hesperetin has been increased by 228.36 times, which was a natural flavonoid compound with antioxidant effect and was widely used in fruits, flowers and food (48). The hesperetin in millet yogurt is mainly derived from the added millet enzymatic fermentation broth, which is also consistent with the high antioxidant capacity of the previous yogurt. N-Acetylornithine is an intermediate from L-glutamic acid to L-arginine enzymatic biosynthesis, and the content was increased 271.22 times. L-methionine can be used in synthetic vitamins, which can protect the liver, and it is an essential amino acid, which has significant value to the human body (49). L-methionine was increased 55.67 times in non-volatile metabolites. Most flavored compounds in yogurt were because of creamy fat decomposition and microbial transformation of lactose and citrate. The addition of millet enzymatic hydrolysis fermentation broth to yogurt enriched the variety of flavoring substances in yogurt, and this practice could also counteract the natural sourness of yogurt. This was consistent with previous reports that the addition of plant or fruit components might add sweetness or nutritional value of yogurt (50). Compared with ordinary yogurt, adding F8 and millet could improve the flavor and yogurt nutrients. The results of this part of the experiment provide insights into the metabolic mechanisms of *Lactiplantibacillus plantarum subsp. plantarum* F8, mainly related to the biosynthesis of secondary metabolites, amino acid metabolism and nucleotide metabolism. As well as the fingerprinting of the effect of millet enzymatic digest on non-volatile flavor substances in yogurt represents a real advance in the study of metabolites in yogurt.

### Analysis of Volatile Flavor Compounds in Yogurt by HS-SPME-GC-MS

**Figure 8:**
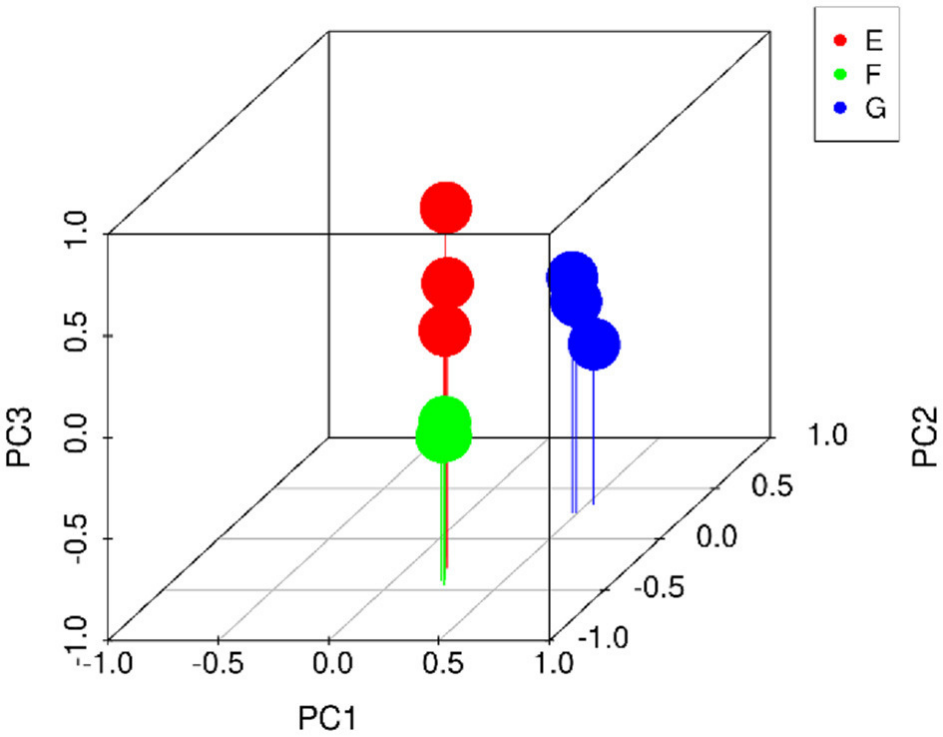
3D PCA volatile metabolite score was used to compare traditional starter (Y), traditional starter and *Lactiplantibacillus plantarum subsp. plantarum F8* (Y-F8), and millet yogurt (Y-F8-M) (Red circle: Y, green circle: Y-F8, blue circle: Y-F8-M).

PCA exhibited multiple components with large-to-small contributions (PC1, PC2, PC3…). 3D PCA used the first three dimensions for mapping to observe the distribution of samples in three-dimensional space. As indicated in Figure 8, the sample repeatability of the three yogurt groups was good, and the differences among the groups were large. The distance between group E and group F was relatively close, indicating that the addition of *Lactiplantibacillus plantarum subsp. plantarum* F8 affected the volatile flavor compounds of the two yogurts. In contrast, the distance among group G, group F and group E were far, indicating that the amount of added millet remarkably contributed to the volatile flavor of yogurt.

**Figure 9:**
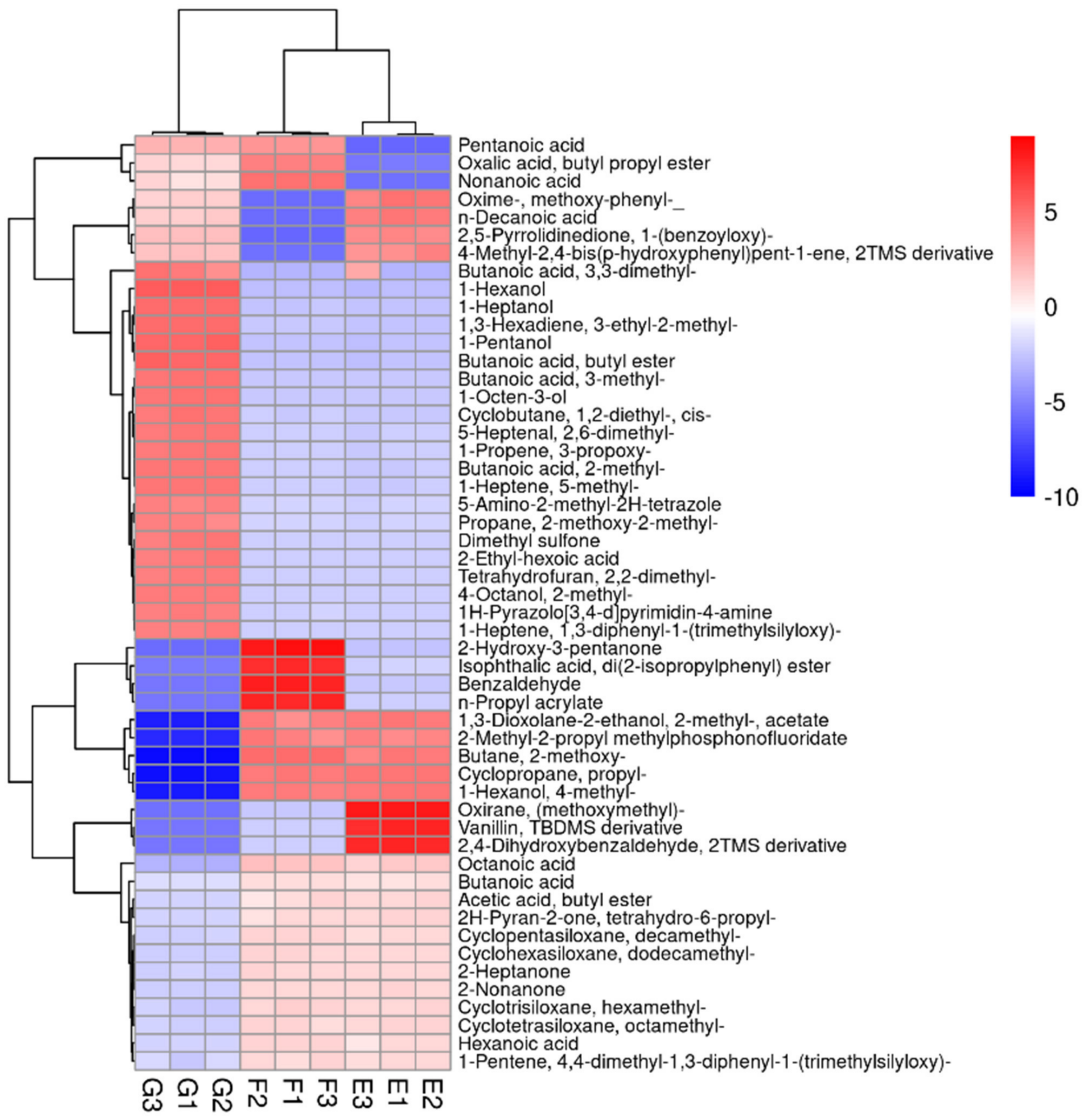
Heat-map and hierarchical clustering of volatile metabolite profiles in yogurt. The normalized volatile metabolite abundance was visualized by color: red indicates the highest values, and blue indicates the lowest values (E: Y, F: Y-F8, G: Y-F8-M).

From Figure 9, it can be seen that 54 volatile flavor substances were found in yogurt, including alcohols, lipids, organic acids and hydrocarbons. Compared with group E, 12 volatile flavor substances were upregulated, and seven were downregulated in group F. Among these volatile flavor substances, Nonanoic acid was produced in *Lactiplantibacillus plantarum subsp. plantarum* via the fatty acid metabolic pathway and has a fruity flavor (3). Nonanoic acid was also detected and significantly upregulated when Tian studied the effect of four kinds of probiotic LAB on fermented milk flavor substances (51). This indicated that Nonanoic acid might be a typical flavor substance produced by probiotic LAB fermented yogurt. Oxalic acid, butyl propyl ester was produced by esterification in *Lactiplantibacillus plantarum subsp. plantarum* and has an aromatic odor, which was an important component of yogurt aroma (3). 2-Hydroxy-3-pentanone was produced by the conversion of glycerol by *Lactiplantibacillus plantarum subsp. plantarum* through the glycolytic pathway and was commonly used in food flavoring agents (52). Benzaldehyde is an organic compound formed when the hydrogen of benzene is replaced by an aldehyde group. It was the most commonly used aromatic aldehyde with a bitter almond, cherry and nutty flavor (53). The current methods of producing benzaldehyde were mainly chemical synthesis or extraction from plants (51). This strain of *Lactiplantibacillus plantarum subsp. plantarum* produced benzaldehyde at relatively high levels and might have the potential to produce benzaldehyde using microbial fermentation. Therefore, the addition of *Lactiplantibacillus plantarum subsp. plantarum* F8 might have a particularly positive effect on the abundance of volatile flavor substances in yogurt.

Compared with group F, 25 volatile flavor substances were upregulated in group G, of which the content of 5-Heptenal, 2,6-dimethyl (with grassy, sweet, cucumber and watermelon aroma), 2-Ethyl-hexoic acid (generally used in barbecues, beverages and sweets and naturally found in wine and beer), 2-Ethyl-hexoic acid (slightly smelly, used for synthetic spices), 1-Hexanol (with fruity aroma), Butanoic acid, butyl ester (with fresh sweet fruit aroma, similar to banana and pineapple flavor), 1-Pentanol (slightly fruity), 1-Heptanol (with fresh and light oily fat aroma, similar to whiskey and wine-like aroma), n-Decanoic acid (with a rancid taste), Butanoic acid, and 3-methyl (with a strong cheese aroma) was significantly increased (17, 54, 55). The formation of yogurt flavor is a complex and dynamic biochemical process (56). Millet enzymatic fermentation broth were added to enrich the volatile flavor substances in yogurt, mask the production of unpleasant volatile flavor substances, counteract the natural sour taste in yogurt, and make the product more in line with consumer taste. The addition of *Lactiplantibacillus plantarum subsp. plantarum* F8 and the plant-based ingredient millet may make an essential contribution to the flavor of yogurt. Additionally, they promote the formation of volatile metabolites that positively influence the aroma quality of yogurt samples (57). The results of this work provide novel knowledge about the contribution of the isolated strains and the millet enzymatic digests to the flavor profile of yogurt, which will help improve the sensory features of the final product.

## Conclusions

In this study, LAB, namely, *Lactiplantibacillus plantarum subsp. plantarum* F8, was screened, with a SOD yield of 2476.21 ± 1.52 U g ^−1^. Meanwhile, this strain exhibited great potential as a probiotic. When this novel yogurt was used in the millet-based yogurt fermentation, its SOD activity was 19.827 ± 0.323 U mL^−1^, higher than Bright Dairy 1911, Grand Park Old Yogurt and Weigang Flavored Yogurt Ordinary Yogurt. The addition of *Lactiplantibacillus plantarum subsp. plantarum* F8 significantly upregulated 12 non-volatile metabolites in yogurt, and the addition of millet enzymatic fermentation increased the non-volatile metabolites in yogurt. Thirty-seven species were significantly upregulated, including 2-Phenylethanol, Hesperetin, N-Acetylornithine and L-methionine were upregulated by 3169.6, 228.36, 271.22, and 55.67 times, respectively. The addition of *Lactiplantibacillus plantarum subsp. plantarum* F8 had a specific positive effect on the abundance of volatile flavor substances in yogurt. The addition of millet enzymatic fermentation broth enriched the volatile flavor substances in yogurt. The production of unpleasant volatile flavor substances was masked, making the product more in line with consumer taste.

## Data Availability Statement

The original contributions presented in the study are included in the article/supplementary material, further inquiries can be directed to the corresponding author/s.

## Author Contributions

XF: writing—original draft, data curation, investigation, methodology, and formal analysis. XL: conceptualization, writing—review and editing, data curation, formal analysis, investigation, and project administration. DP: conceptualization, project administration, funding acquisition, validation, and supervision. TZ: data curation, methodology, and supervision. ZW: formal analysis, visualization, resources, and methodology. YG: resources and methodology. XZ: conceptualization and project administration. ZS: conceptualization and formal analysis. All authors contributed to the article and approved the submitted version.

## Funding

This work was financially supported by Natural Science Funding of China (31972048), Jiangsu Agricultural Science and Technology Innovation Fund [CX(18)3036], Jiangsu Science and Technology Department (BE2018397), the Scientific Research Foundation of Graduate School of Ningbo University (IF2021084).

## Conflict of Interest

The authors declare that the research was conducted in the absence of any commercial or financial relationships that could be construed as a potential conflict of interest.

## Publisher's Note

All claims expressed in this article are solely those of the authors and do not necessarily represent those of their affiliated organizations, or those of the publisher, the editors and the reviewers. Any product that may be evaluated in this article, or claim that may be made by its manufacturer, is not guaranteed or endorsed by the publisher.
